# Association between syndesmophyte and metabolic syndrome in patients with psoriatic arthritis or ankylosing spondylitis: a cross-sectional study

**DOI:** 10.1186/s12891-021-04222-8

**Published:** 2021-04-20

**Authors:** Chonachan Petcharat, Varalak Srinonprasert, Praveena Chiowchanwisawakit

**Affiliations:** 1 Division of Rheumatology, Department of Medicine, Faculty of Medicine Siriraj Hospital, Mahidol University, 2 Wanglang Road, Bangkoknoi, Bangkok, 10700 Thailand; 2 Division of Geriatric Medicine, Department of Medicine, Faculty of Medicine Siriraj Hospital, Mahidol University, Bangkok, Thailand

**Keywords:** Prevalence, Factors, Metabolic syndrome, Psoriatic arthritis, Ankylosing spondylitis, Cross-sectional study

## Abstract

**Objective:**

To investigate the prevalence of and factors associated with metabolic syndrome (MetS) in patients with psoriatic arthritis (PsA) or ankylosing spondylitis (AS).

**Methods:**

This cross-sectional study included PsA or AS patients who attended Siriraj Hospital (Bangkok, Thailand) during March 2014 to October 2017. The Harmonized MetS definition was used to diagnose MetS. Demographic, clinical, and spinal radiographic data were collected. Logistic regression was used to identify factors associated with MetS.

**Results:**

Among 319 patients, 153 had AS and 166 had PsA. MetS was present in 43% of PsA and 19% of AS (*p* < 0.001). Multiple regression analysis identified body mass index (BMI) > 23 (odds ratio [OR]: 3.7), female gender (OR range: 3.8–3.9), and the number of syndesmophytes or ankylosis [SynAnk] (OR: 1.1) were associated with MetS among PsA patients. For AS patients, BMI > 23 (OR: 9.1) and age > 40 (OR: 4.3) were associated with MetS. Disease activity index was not associated with MetS.

**Conclusions:**

MetS was significantly more prevalent in PsA than in AS. Structural change of the spine was associated with MetS in PsA. PsA patients with being female, BMI > 23 or evidence of spinal change should be informed to screen for MetS. AS patients with age > 40 or BMI > 23 should be informed to screen for MetS.

## Introduction

Spondyloarthritis (SpA) comprises a group of chronic inflammatory rheumatic diseases, including ankylosing spondylitis (AS) and psoriatic arthritis (PsA) [1]. Chronic systemic inflammation and cardiovascular disease (CVD) [2] are thought to have a common inflammatory pathogenesis that is not clearly understood. Metabolic syndrome (MetS) is defined as a cluster of classical and modifiable cardiovascular risk factors, including insulin resistance, central obesity, elevated blood pressure, high triglyceride levels, and low levels of high-density lipoprotein cholesterol (HDL-C) [3]. Individuals with MetS are at increased risk of developing CVD over the following 5 to 10 years [3]. MetS may also be an important risk factor for CVD in patients with chronic inflammatory arthritis [4]. Chronic inflammatory joint disease, especially rheumatoid arthritis, may also be an important factor that relates to CVD [5]. Subclinical atherosclerotic disease has been reported in patients with PsA or AS [6, 7].

Published systematic reviews and meta-analyses concluded that the prevalence of CVD is increased in PsA patients (odds ratio [OR]: 1.6) [8], and in AS patients (OR: 1.5–1.6) [9] when compared with the general population. However, heterogeneous study designs and varying cardiovascular outcome criteria raise concerns about potential biases [8–10]. The increased risk of CVD in PsA and AS may be due to chronic joint inflammation, traditional risk factors for CVD, or both [11]. Furthermore, chronic inflammatory joint disease and MetS are believed to share a common inflammatory pathogenesis [12, 13].

Several studies reported the prevalence of MetS to be higher in patients with PsA (38.0 to 59.0%) than in general population [10, 14–17]. Similarly, other studies reported the prevalence of MetS in patients with AS to be 11 to 45% [15, 18, 19]. This observed variability in prevalence estimates may be explained by the use of different MetS definitions, ethnicities, lifestyles, types of inflammatory disease, and treatments for chronic rheumatic disease. There have been few published reports focusing on MetS in PsA patients and AS patients in Asia, and there have been none from Thailand. Accordingly, the aim of this study was to investigate the prevalence of and factors associated with MetS in patients with PsA or AS at Siriraj Hospital – Thailand’s largest national tertiary referral center.

## Materials and methods

### Patients

This cross-sectional study enrolled patients older than 18 years that were diagnosed with AS according to Modified New York criteria [20], or diagnosed with PsA according to the classification criteria for psoriatic arthritis [21], and that had visited the outpatient clinics of the Faculty of Medicine Siriraj Hospital, Mahidol University, Bangkok, Thailand during the 15 March 2014 to 31 October 2017 study period. Siriraj Hospital is a 2300-bed national tertiary center, and Thailand’s largest teaching hospital. Patients unwilling to be assessed for MetS and those with symptomatic infection were excluded. Written informed consent was obtained from all participants. The protocol for this study was approved by the Siriraj Institutional Review Board (SIRB) (COA no. Si 147/2014) on 14 March 2014, and complied with the Declaration of Helsinki 1964 and all of its later amendments.

### Clinical and laboratory assessments

Demographic data, disease duration, history of comorbidities (hypertension, diabetes, dyslipidemia, cardiovascular disease, and cerebrovascular disease), occupation, smoking status (current smoker, ex-smoker, or never-smoked), current use of non-steroidal anti-inflammatory drugs (NSAIDs) and/or disease modifying anti-rheumatic drugs (DMARDs), current habitual exercise, and scores from the Thai versions of patient self-reported outcome assessments were collected, recorded, and analyzed. The aforementioned outcome assessments included the Bath Ankylosing Spondylitis Disease Activity Index (BASDAI) [22], Patient Global Assessment (PGA), and the Patient Pain (PP) assessment, which is a numerical scale from 0 (no pain) to 10 (very severe pain). Occupation was divided into physical and non-physical work.

Physical examination of peripheral joints (66 swollen/68 tender joint counts) and dactylitis were assessed by one rheumatologist (PC or CP). Body weight, height, waist circumference (WC), and blood pressure (BP) were collected. WC was measured at the midpoint between the lower margin of the last rib and the top of the iliac crest while standing, and at the end of normal expiration. If elevated BP (systolic > 130 and/or diastolic > 85 mmHg) was identified, participants were asked to rest in a seated position for 10 min, and then the BP measurement was repeated. All physical examinations were performed regardless of questionnaire and blood test results. Blood was drawn after 12 h of fasting to determine fasting glucose (FG), triglycerides (TG), HDL-C, and C-reactive protein (CRP). CRP was tested by particle-enhanced immunoturbidimetric assay (cobas c502 module, cobas 8000 modular analyzer series, Roche Diagnostics International Ltd., Rotkreuz, Switzerland) (normal range: 0–5 mg/L).

Body mass index (BMI) was calculated as weight/height^2^ (kg/m^2^), and 23 kg/m^2^ (BMI > 23) was determined to be the threshold for overweight status in Asian population [23]. The Ankylosing Spondylitis Disease Activity Score–C-reactive protein (ASDAS-CRP) calculation included PGA, BASDAI questions 2, 3, and 6, and CRP [24]. The Disease Activity Index for Psoriatic Arthritis (DAPSA) was based on the summation of 68 tender joint count (TJC), 66 swollen joint count (SJC), PGA, PP, and CRP (mg/dl) [25]. The Clinical Disease Activity Index for Psoriatic Arthritis (cDAPSA) included all of items in the DAPSA with the exception of CRP [26].

Lateral views of cervical and lumbar spine radiographs taken within 2 years were included. The presence or absence of syndesmophyte or ankylosis at each corner from lower corner of the second cervical vertebrae to the upper corner of the first thoracic spine vertebrae, and from lower corner of the twelfth thoracic vertebrae to the upper corner of the first sacral spine was recorded. Syndesmophyte was defined as bone growth originating from the vertebral endplate in the anterior one-quarter of the discovertebral space, or from the anterior vertebral cortex [27]. Ankylosis was defined as the presence of bridging between the anterior vertebral cortices or between vertebral end plates in the anterior quarter of the discovertebral space [27]. The assessment for syndesmophyte and ankylosis was performed by an experienced rheumatologist (PC) who was blinded to patient clinical data, who completed the Spondyloarthritis Radiography training module (http://www.carearthritis.com/spar/), and who had an excellent intraclass correlation coefficient with expert #1 (0.98, 95% confidence interval [CI]; 0.96–0.99), and expert #2 (0.93, 95% CI: 0.82–0.97).

### Definition of metabolic syndrome

The presence of MetS was determined according to the Harmonized definition (2009) [3] that assigns the diagnosis if a patient meets at least three of the following five criteria: 1) WC > 90 cm in males or > 80 cm in females; 2) SBP > 130 and/or diastolic blood pressure (DBP) > 85 mmHg, or receiving antihypertensive drug treatment in a patient with a history of hypertension; 3) elevated FG > 100 mg/dL or receiving drug treatment for elevated glucose; 4) elevated TG > 150 mg/dL or receiving drug treatment for elevated triglycerides (fibrates, nicotinic acid, high-dose omega-3 fatty acids); and/or, 5) reduced HDL-C < 40 mg/dL in males or < 50 mg/dL in females, or receiving drug treatment for reduced HDL-C (fibrates, nicotinic acid).

### Statistical analysis

Continuous variables were compared using Student’s t-test or Mann-Whitney U test, and those results are shown as mean ± standard deviation for normally distributed data or median and interquartile range for non-normally distributed data. Categorical variables were compared using Pearson’s χ^2^ test or Fisher’s exact test, and those results are given as frequency and percentage. A *p-*value of less than 0.05 was considered statistically significant. Imputation was not conducted for missing data. Patients with an ASDAS-CRP score of 2.1 or greater (ASDAS-CRP ≥2.1) were considered to have a high level of disease activity [24]. A DAPSA score of more than 14 (DAPSA > 14), and a cDAPSA score of more than 13 (cDAPSA > 13) were considered to have moderate to high disease activity [28].

Univariate logistic regression analysis was used to determine association between evaluated factors and MetS. Variables with a *p*-value less than 0.2 and variables of interest, including smoking, disease duration, and disease activity index, were included in multiple regression analysis. Enter method was applied in fitting a multiple logistic regression model to determine effect of each factor adjusted for other factors in the model. To prevent multicollinearity in multiple regression analysis, only one disease activity index (ASDAS-CRP, DAPSA, or cDAPSA) was included in each model and multicollinearity was checked using variance inflation factor of less than 10. Statistical analyses were performed using SPSS 17.0 software (SPSS, Inc., Chicago, IL, USA).

## Results

### Study population characteristics

A total of 319 (153 AS and 166 PsA) participants were recruited for this study. The mean age of patients was 45.5 ± 12.2 years, and 148 (46.4%) were female. Two patients with PsA did not have a BASDAI assessment. A total of 295 (146 AS and 149 PsA) patients had spinal imaging, and 246 (114 AS, 132 PsA) patients had CRP performed. Fifteen (4 AS and 11 PsA) patients were treated with tumor necrotic factor alpha inhibitor (TNFi). Eight (4 AS and 4 PsA) patients were treated with oral prednisolone. PsA patients were significantly older, had higher BMI, and were more likely to be female, have a history of hypertension, have type 2 diabetes mellitus, have dyslipidemia, have occupational physical work, and have a shorter disease duration than AS patients (Table 1). The proportion of smoking, current NSAID use, the mean ASDAS-CRP, the median number of spinal corners having syndesmophyte or ankylosis per patient (SynAnk), and current DMARDs use were similar between patients with PsA and AS.

**Table 1:** Participant characteristics compared between the ankylosing spondylitis and psoriatic arthritis groups

Characteristics	All (***n*** = 319)	Ankylosing spondylitis (***n*** = 153)	Psoriatic arthritis (***n*** = 166)	***p***-value†
Age (yrs), mean ± SD	45.5 ± 12.2	41.7 ± 11.2	49.0 ± 12.1	***< 0.001***
Female gender, n (%)	148 (46.4%)	59 (38.6%)	89 (53.6%)	***0.007***
Disease duration (yrs), median (IQR)	5.0 (8.0)	10.0 (15.0)	3.0 (5.8)	***< 0.001***
Hypertension, n (%)	71 (22.3%)	25 (16.3%)	46 (27.7%)	***0.015***
Diabetes, n (%)	34 (10.7%)	6 (3.9%)	28 (16.9%)	***< 0.001***
Dyslipidemia, n (%)	54 (16.9%)	18 (11.8%)	36 (21.7%)	***0.018***
Current smoker, n (%)	27 (8.5%)	12 (7.8%)	15 (9.0%)	0.702
Ex-smoker, n (%)	28 (8.8%)	10 (6.5%)	18 (10.8%)	0.240
Current exercise, n (%)	134 (42.0%)	70 (45.8%)	64 (38.6%)	0.193
Occupational physical work, n (%)	122 (38.2%)	49 (32.0%)	73 (44.0%)	***0.028***
BMI (kg/m^2^), mean ± SD	24.4 ± 4.9	23.4 ± 4.9	25.3 ± 4.7	***0.001***
ASDAS-CRP, mean ± SD	2.1 ± 1.1	2.1 ± 1.1	1.9 ± 1.1	0.993
No. of SynAnk, median (IQR)	3.0 (8.5)	2.5 (11.0)	4.0 (8.0)	0.755
Current DMARDs, median (IQR)	1.0 (0)	1.0 (1.0)	1.0 (1.0)	0.424
Current NSAIDs, n (%)	115 (36.1%)	34 (33.7%)	81 (37.2%)	0.546

A *p*-value < 0.05 indicates statistical significance

†Reflects comparison between ankylosing spondylitis and psoriatic arthritis

**Abbreviations:** *SD* Standard deviation; *IQR* Interquartile range; *BMI* Body mass index; *ASDAS-CRP* Ankylosing Spondylitis Disease Activity - C-reactive protein; *SynAnk* Syndesmophyte or ankylosis; *DMARDs* Disease-modifying antirheumatic drugs; *NSAIDs* Non-steroidal anti-inflammatory drugs

### Prevalence of MetS and characteristics of patients with MetS

Patients with PsA had significantly higher proportions of all MetS components, including increased WC (*p* = 0.001), elevated BP (*p* < 0.001), elevated FG (*p* < 0.001), elevated TG (*p* = 0.01), and decreased HDL-C (*p* = 0.016) when compared to AS patients (Table 2). The prevalence of MetS was 31.7% for all participants, 43.4% for patients with PsA, and 19% among patients with AS (*p* < 0.001). Five patients with PsA had personal history of CVD, and two of those had MetS.

**Table 2:** Characteristics compared between those with and without metabolic syndrome further compared among all included patients, ankylosing spondylitis patients, and psoriatic arthritis patients

Characteristics	All (***n*** = 319)		Ankylosing spondylitis (***n*** = 153)		Psoriatic arthritis (***n*** = 166)	
	Metabolic syndrome		Metabolic syndrome		Metabolic syndrome	
	Yes (***n*** = 101)	No (***n*** = 218)	Yes (***n*** = 29)	No (***n*** = 124)	Yes (***n*** = 72)	No (***n*** = 94)
Number of MetS criteria, median (IQR)	4.0 (1.0)	1.0 (2.0)*	4.0 (1.0)	1.0 (2.0)*	3.5 (1.0)	1.0(2.0)*
Elevated WC, n (%)	93 (92.1%)	60 (27.5%)*	24 (82.8%)	34 (27.4%)*	69 (95.8%)	26 (27.7%)*
Elevated BP, n (%)	87 (86.1%)	55 (25.2%)*	24 (82.8%)	28 (22.6%)*	63 (87.5%)	27 (28.7%)*
Elevated FG, n (%)	74 (73.3%)	38 (17.4%)*	23 (79.3%)	13 (10.5%)*	51 (70.8%)	25 (26.6%)*
Elevated TG, n (%)	50 (49.5%)	20 (9.2%)*	12 (41.4%)	12 (9.7%)*	38 (52.8%)	8 (8.5%)*
Reduced HDL-C, n (%)	63 (62.4%)	26 (11.9%)*	21 (72.4%)	12(9.7%)*	42 (58.3%)	14 (14.9%)*
Age (yrs), mean ± SD	52.3 ± 10.1	42.3 ± 11.9*	48.0 ± 9.2	40.3 ± 11.2*	54.0 ± 9.9	45.1 ± 12.3*
Female gender, n (%)	55 (54.5%)	93 (42.7%)*	8 (27.6%)	51 (41.1%)	47 (65.3%)	42 (44.7%)*
History of smoking, n (%)	13 (12.9%)	42 (19.3%)	4 (13.8%)	18 (14.5%)	9 (12.5%)	24 (25.5%)*
Current smoker, n (%)	5 (5.0%)	22 (10.1%)	3 (10.3%)	9 (7.3%)	2 (2.8%)	13 (13.8%)*
Current exercise, n (%)	40 (39.6%)	94 (43.1%)	9 (31.0%)	40 (49.2%)	31 (43.1%)	33 (35.1%)
Physical work, n (%)	37 (36.6%)	85 (39.0%)	8 (27.6%)	41 (33.1%)	29 (40.3%)	44 (46.8%)
BMI (kg/m^2^), mean ± SD	27.2 ± 6.0	23.1 ± 4.5*	27.0 ± 4.5	22.5 ± 4.6*	27.3 ± 4.5	23.7 ± 4.3*
Disease duration (yrs), median (IQR)	5.0 (8.0)	5.0 (9.0)	10.0(14.5)	10(13.8)	3.0(6.5)	3.0(4.6)
ASDAS-CRP, mean (SD)	2.0 (1.1)	2.2 (1.1)	2.0 (1.1)	2.2(1.1)	2.0 (1.1)	2.3 (1.2)
DAPSA, median (IQR)	N/A	N/A	N/A	N/A	6.3 (10.5)	9.1 (12)
cDAPSA, median (IQR)	N/A	N/A	N/A	N/A	6.0(9.5)	8.0 (11)
No. of SynAnk, median (IQR)	5.5 (8.0)	2.0 (7.0)*	5.0 (11.5)	2.0 (10.0)*	6.0 (9.0)	2.0 (5.0)*
Current DMARDs, median (IQR)	1.0 (1.5)	1.0 (0)	1.0 (1.5)	1.0 (1.0)	1.0 (1.0)	1.0 (0.25)
Current NSAIDs, n (%)	34 (33.7%)	81 (37.2%)	12 (41.3%)	53 (42.7%)	22 (30.6%)	28 (29.8%)

*Indicates a *p*-value< 0.05

**Abbreviations:** *MetS* Metabolic syndrome; *IQR* Interquartile range; *WC* Waist circumference; *BP* Blood pressure; *FG* Fasting glucose; *TG* Triglycerides; *HDL-C* High-density lipoprotein cholesterol; *SD* Standard deviation; *BMI* Body mass index; *ASDAS-CRP* Ankylosing Spondylitis Disease Activity - C-reactive protein; *cDAPSA* Clinical Disease Activity Index for Psoriatic Arthritis; *DAPSA* Disease Activity Index for Psoriatic Arthritis; *SynAnk* Syndesmophyte or ankylosis; *DMARDs* Disease modifying antirheumatic drugs; *NSAIDs* Non-steroidal anti-inflammatory drugs, *N/A* Not applicable

Among all study participants, patients with MetS were more likely to be older, female, to have higher BMI, and to have a higher number of SynAnk than those without MetS. Disease duration, ASDAS-CRP, the number of current DMARDs, and the proportion of NSAIDs uses were not significantly different between those with and without MetS. Many of the same associations were observed in subgroup analysis that were observed when we analyzed data from the entire study cohort. However, in the PsA subgroup, participants that smoked were less likely to have MetS, and gender was not associated with MetS in patients with AS.

### Factors associated with MetS in patients with PsA

Regarding PsA patients, age > 40 (*p* < 0.001), BMI > 23 (*p* < 0.001), and the number of SynAnk (*p* = 0.001) were associated with MetS in univariate analysis. The disease activity index [cDAPSA > 13 (*p* = 0.331), DAPSA > 14 (*p* = 0.230)] was not associated with MetS (Table 3). However, female gender (*p* = 0.009) and smoking status (*p* = 0.041) were also significantly associated with MetS in PsA patients. Factors that were significant in univariate analysis were further analyzed in multiple regression analysis. BMI > 23 (*p* = 0.008) and female gender (*p* = 0.007) were the two strongest associations with MetS. PsA patients with a higher number of SynAnk were more likely to be MetS (*p* = 0.002). DAPSA was not associated with MetS (*p* > 0.05).

**Table 3:** Univariate and multiple logistic regression analyses for factors significantly associated with metabolic syndrome in patients with psoriatic arthritis

Factors	Univariate analysis		Multiple regression analysis	
	OR	95% CI	OR	95% CI
Age > 40 years	5.41*	2.12–13.85	2.98	0.93–9.50
Female gender	2.33*	1.24–4.38	3.85*	1.45–10.22
History of smoking	0.42*	0.18–0.96	0.54	0.16–1.83
BMI > 23 kg/m^2^	4.04*	1.92–8.48	3.67*	1.40–9.60
Disease duration ≥10 years	1.63	0.74–3.61	1.64	0.57–4.74
No. of SynAnk	1.12*	1.05–1.19	1.14*	1.05–1.23
cDAPSA > 13	0.69	0.33–1.46	N/A	N/A
DAPSA > 14	0.62	0.29–1.35	0.42	0.16–1.10
ASDAS-CRP > 2.1	0.61**	0.3–1.24	N/A	N/A

*Indicates a *p*-value < 0.05; **Indicates a *p*-value from 0.05 to < 0.2

**Abbreviations:** *OR* Odds ratio; *CI* Confidence interval; *BMI* Body mass index; *SynAnk* Syndesmophyte or ankylosis; *cDAPSA* Clinical Disease Activity Index for Psoriatic Arthritis; *DAPSA* Disease Activity Index for Psoriatic Arthritis; *ASDAS-CRP* Ankylosing Spondylitis Disease Activity – C-reactive protein; *N/A* Not applicable

### Factors associated with MetS in patients with AS

In univariate analysis, MetS was significantly associated with age > 40 and BMI > 23. Conversely, gender, smoking, disease duration, the number of SynAnk, and disease activity were not associated with MetS (Table 4). In multiple regression analysis, only BMI > 23 (*p* < 0.001) and age > 40 (*p* = 0.01) were significantly and independently associated with MetS in AS patients.

**Table 4:** Univariate and multiple logistic regression analyses for factors significantly associated with metabolic syndrome in patients with ankylosing spondylitis

Variables	Univariate analysis		Multiple regression analysis	
	OR	95% CI	OR	95% CI
Age > 40	4.09*	1.56–10.73	4.31*	1.43–13.01
Female gender	0.55	0.22–1.33	N/A	N/A
History of smoking	0.94	0.29–3.03	N/A	N/A
BMI > 23 kg/m^2^	8.51*	3.03–23.89	9.09*	3.13–26.36
Disease duration > 10 years	0.88	0.54–1.44	N/A	N/A
No. of SynAnk	1.04**	0.99–1.09	1.0	0.94–1.06
ASDAS-CRP > 2.1	0.57	0.23–1.45	N/A	N/A

*Indicates a *p*-value < 0.05; **Indicates a *p*-value from 0.05 to < 0.2

**Abbreviations:** *OR* Odds ratio; *CI* Confidence interval; *BMI* Body mass index; *SynAnk* Syndesmophyte or ankylosis; *ASDAS-CRP* Ankylosing Spondylitis Disease Activity - C-reactive protein; *N/A* Not applicable

### Factors associated with MetS stratified by gender

Interestingly, female patients with PsA had a significantly higher prevalence of MetS [47 (52.8%) vs. 25 (32.5%), *p* = 0.008] and a lower median [inter quartile range (IQR)] number of SynAnk [2.0 (8.0) vs. 5.0 (6.5), *p* = 0.006] than male patients with PsA. Conversely, the prevalence of MetS in male patients with AS (22.3%) was higher than in female patients (13.6%) (*p* = 0.177). Men with AS also had a significantly higher median (IQR) number of SynAnk [4 (15) vs. 0 (4), *p* < 0.001] and a longer median (IQR) disease duration [10 (14.0) vs. 6.0 (9.0) years, *p* = 0.006] than women with AS. As a result, variables of interest from our analysis of the entire study cohort were further analyzed stratified by gender.

In the female subgroup, age > 40, PsA, BMI > 23, and the number of SynAnk were significantly associated with MetS, while ASDAS-CRP > 2.1 had marginal association in univariate analysis (Table 5). Conversely, disease duration > 10 years and smoking were not associated with MetS. In multiple regression analysis, PsA (*p* = 0.028), BMI > 23 (*p* = 0.014), and the number of SynAnk (*p* = 0.005) were significantly associated with MetS, while age > 40 and ASDAS-CRP > 2.1 were not significantly associated with MetS.

**Table 5:** Univariate and multiple logistic regression analyses for factors significantly associated with metabolic syndrome compared between genders among all patients included in this study

Variables	Characteristics Metabolic syndrome		Univariate analysis		Multiple regression analysis	
	Yes	No	OR	95% CI	OR	95% CI
**Female gender, n (%)**	55 (37.2%)	93 (62.8%)				
Psoriatic arthritis, n (%)	47 (85.5%)	42 (45.2%)*	7.42*	3.13–17.57	3.48*	1.14–10.60
Age > 40, n (%)	50 (90.9%)	48 (51.6%)*	9.96*	3.62–27.41	2.44	0.95–12.48
History of smoking, n (%)	2 (3.6%)	2 (2.2%)	1.54	0.21–11.24	N/A	N/A
BMI > 23 kg/m^2^, n (%)	42 (76.4%)	40 (43.0%)*	4.5*	2.12–9.58	3.74*	1.31–10.60
Disease duration > 10 years, n (%)	12 (21.8%)	27 (29.0%)	0.68	0.31–1.49	N/A	N/A
SynAnk, median (IQR)	5 (7)	0 (3)*	1.15*	1.06–1.25	1.18*	1.05–1.32
ASDAS-CRP > 2.1, n (%)	15 (34.1%)	36 (52.9%)**	0.36*	0.16–0.81	0.37	0.14–1.01
**Male gender, n (%)**	46 (26.9%)	125 (73.1%)				
Psoriatic arthritis, n (%)	25 (54.3%)	52 (41.6%)	1.83**	0.91–3.68	0.96	0.43–2.17
Age > 40, n (%)	39 (84.8%)	75 (60.0%)*	3.47*	1.43–8.45	2.91*	1.07–7.93
History of smoking, n (%)	11 (23.9%)	40 (32.0%)	0.65	0.29–1.47	N/A	N/A
BMI > 23 kg/m^2^, n (%)	42 (91.3%)	56 (45.5%)*	13.27*	4.45–39.56	11.42*	3.75–34.79
Disease duration > 10 years, n (%)	23 (50.0%)	55 (44.0%)	1.27	0.65–2.51	N/A	N/A
SynAnk, median (IQR)	7.5 (11)	4.0 (11)*	1.05*	1.01–1.10	1.02	0.97–1.08
ASDAS-CRP > 2.1, n (%)	13 (34.2%)	43 (44.8%)	0.73	0.33–1.61	N/A	N/A

*Indicates a *p*-value < 0.05; **Indicates a *p*-value from 0.05 to < 0.2

**Abbreviations:** *OR* Odds ratio; *CI* Confidence interval; *BMI* Body mass index; *SynAnk* Number of syndesmophyte or ankylosis; *ASDAS-CRP* Ankylosing Spondylitis Disease Activity - C-reactive protein; *N/A* Not applicable

Regarding univariate analysis in male subgroup, BMI > 23, age > 40, and the number of SynAnk were significantly associated with MetS, while PsA had marginal association. ASDAS-CRP > 2.1, disease duration > 10 years, and smoking were not associated with MetS. In multiple regression analysis, only BMI > 23 (*p* < 0.001) and age > 40 (*p* = 0.037) were significantly associated with MetS,

## Discussion

Several studies have reported association between PsA and MetS [10, 14–16, 29], while the relationship between MetS and AS is not as strong [15, 19, 30]. We observed a high prevalence of MetS in Thai patients with PsA (43.4%). In contrast, the prevalence of MetS in AS patients was 19.0%, which is comparable to the prevalence rate in the 35–45 age group (10–20% for men, and 23% for women); however, it is considerably lower than the 32.6% prevalence of MetS in overall general population in Thailand [31]. A study from Hong Kong reported a prevalence of MetS was increased in patients with PsA of 38% while it was not increased in patients with AS [15].

BMI is significantly associated with MetS in the general population [32]. BMI was also found to be strongly associated with MetS in our patients with AS or PsA, which is consistent with other published findings [15, 17]. Age was associated with MetS only in patients with AS in the present study, which is similar to the finding of Papadakis, et al. [18]. The association between age and MetS in PsA is unclear [16, 33]. Increasing age and female gender were reported to be associated with MetS in the general population in Europe [34] and in Thailand [31]. Interestingly, female gender was associated with MetS in PsA, but not with AS in our study. Thus, we further investigated for association between evaluated factors and MetS stratified by gender. That analysis revealed that PsA patients are more likely to have MetS than AS patients in the female subgroup. In contrast, patients with PsA or AS had a comparable likelihood of having MetS in the male subgroup. Eder, et al. [35] reported the level of leptin, which is an adipokine that is associated with obesity [36], to be higher in female patients with PsA than in those with psoriasis, but it was comparable in men [35]. Gender may affect dysregulation of adipokines differently in each inflammatory disease. Moreover, gender has a different effect on the clinical characteristics and severity of AS [37] and PsA [38]. Males demonstrated more rapid radiographic progression compared to females with AS [37], while male patients with PsA had a more favorable clinical outcome than females [38].

In this study, Thai female patients with PsA had a significantly higher prevalence of MetS (52.8%) than men (32.8%); however, they had a lower proportion of SynAnk. Conversely, the prevalence of MetS in male patients with AS was higher than in female patients, although that difference was not statistically significant. Male patients with AS had longer disease duration and greater structural spinal change than female patients. New bone formation in the spine in AS was associated with resolution of spinal inflammation [39]. Therefore, the presence of SynAnk in AS patients may reflect previous inflammation. A higher number of SynAnk, therefore, suggests a greater degree of previous inflammation. Unfortunately, this study was a cross-sectional study, and our sample of AS patients with MetS was too small to permit further analysis.

The presence of SynAnk was significantly associated with MetS in patients with PsA in the present study. This is similar to a study by Haroon and colleagues that found radiographic damage on peripheral or sacroiliac joints and requirement for tumor necrosis factor inhibitor (TNFi) among PsA to be significantly associated with MetS [17]. Interestingly, disease activity index was not associated with MetS in this study. Active joint inflammation was reported to be associated with decreases in serum lipid levels in rheumatoid arthritis and PsA patients [13, 40, 41]. Highly active disease may also result in weight loss [13]. However, the association between current disease activity and MetS in patients with spondyloarthritis remains controversial [17, 33, 40]. Most studies, including the present study, applied a cross-sectional design, and this makes it difficult to determine the duration of persistent active inflammation. Spondyloarthritis is a chronic inflammatory disease, but inflammation levels may fluctuate [42]. Thus, the presence of structural changes of the spine may be more suitable for determining the long-term course of inflammation than disease status activity index. Consistent with that, in addition to the traditional risk factors for MetS, patients with structural spinal changes were more likely to have MetS among PsA and AS patients in our study.

The association between smoking and MetS is not clear [43]. In our study, smoking was negatively associated with MetS in PsA patients in univariate analysis; however, smoking did not remain statistically significant after adjustment for other variables. Men were more likely to smoke than women among patients with PsA [30 (39%) vs. 3 (3.4%), *p* < 0.001]. Moreover, male gender was found to be strongly negatively associated with MetS in PsA patients after adjustment for other factors, including smoking. Therefore, the association between smoking and MetS in PsA patients only in univariate analysis may be influenced by male gender.

This study has some limitations. First, the cross-sectional design does not permit exploration of changes in disease over time. Our measurements of disease activity could not assess the duration of activity of disease over time. Therefore, our results may not reflect the long-term course of the disease, except to the extent that structural changes of the spine are the result of chronic inflammation. Second, we did not have an age- and gender-matched control group. A control group is needed to better understand the contribution of specific risk factors. Third, our study had a small number of AS patients with MetS, which limited our ability to conduct subgroup analysis. Fourth, our participants came from a single tertiary care hospital, so our results may not be generalizable to other care settings and to a larger population of Thai PsA and AS patients. Fifth, some participants did not perform CRP, so there was no ASDAS-CRP or DAPSA assessment. However, our study included other disease status indexes (cDAPSA), and the results were comparable (data not shown). Sixth and last, radiographic change was determined by one rheumatologist who is a well-trained reader. However, the use of two well-trained interpreters may have yielded more accurate assessments of radiographic change. Syndesmophyte and intervertebral ankylosis which were irreversible lesions were counted as radiographic change in this study. They may be less sensitive to detect structural change than the Modified Ankylosing Spondylitis Spine Score. Diffuse idiopathic skeletal hyperostosis (DISH) which is strongly associated with the metabolic syndrome is characterized by chunky bridging osteophyte [44]. An early DISH lesion may be interpreted as syndesmophyte although most lesions can be differentiated. A strength of this study is that we used the Harmonized definition of MetS, which is suitable for use in Asian populations.

## Conclusion

MetS was significantly more prevalent in PsA than in AS. Female, BMI > 23 and structural change of the spine were associated with MetS in PsA. Traditional factors (BMI > 23 and age > 40) were associated with MetS in AS patients. Female patients with PsA were more likely to have MetS than male patients with PsA and female patients with AS. Patients with PsA or AS should be advised to maintain their BMI < 23 kg/m^2^. In addition to traditional factors of MetS, evidence of structural spinal change should be informed to screen for MetS among PsA patients.

## Data Availability

The datasets used and/or analysed during the current study available from the corresponding author on reasonable request.

## Abbreviations

AS: Ankylosing spondylitis
ASDAS-CRP: The ankylosing spondylitis disease activity score– C-reactive protein
BASDAI: The bath ankylosing spondylitis disease activity index
BMI: Body mass index
BP: Blood pressure
cDAPSA: Clinical disease activity index for psoriatic arthritis
CRP: C-reactive protein
CVD: Cardiovascular disease
DAPSA: The disease activity index for psoriatic arthritis
DBP: Diastolic blood pressure
DMARDs: Disease modifying anti-rheumatic drugs
FG: Fasting glucose
HDL-C: High-density lipoprotein cholesterol
MetS: Metabolic syndrome
NSAIDs: Non-steroidal anti-inflammatory drugs
PGA: Patient global assessment
PP: Patient pain
PsA: Psoriatic arthritis
SJC: Swollen joint count
SpA: Spondyloarthritis
SynAnk: Syndesmophyte or ankylosis
TG: Triglycerides
TJC: Tender joint count
WC: Waist circumference
